# Quantification of extracellular volume fraction by cardiac computed tomography for noninvasive assessment of myocardial fibrosis in hemodialysis patients

**DOI:** 10.1038/s41598-020-72417-5

**Published:** 2020-09-21

**Authors:** Akimasa Yamada, Kakuya Kitagawa, Satoshi Nakamura, Masafumi Takafuji, Yoshitaka Goto, Ryuji Okamoto, Kaoru Dohi, Hajime Sakuma

**Affiliations:** 1 Department of Radiology, Mie University Hospital, Tsu, Japan; 2 Department of Advanced Diagnostic Imaging, Mie University Graduate School of Medicine, 2-174 Edobashi, Tsu, Mie 514-8507 Japan; 3 Department of Cardiology, Mie University Hospital, Tsu, Japan

**Keywords:** Cardiology, Nephrology

## Abstract

Extent of myocardial fibrosis in hemodialysis patients has been associated with poor prognosis. Myocardial extracellular volume (ECV) quantification using contrast enhanced cardiac computed tomography (CT) is a novel method to determine extent of myocardial fibrosis. Cardiac CT-based myocardial ECV in hemodialysis patients with those of propensity-matched non-hemodialysis control subjects were compared. Twenty hemodialysis patients (mean age, 67.4 ± 9.6 years; 80% male) and 20 propensity-matched non-hemodialysis controls (mean age, 66.3 ± 9.1 years; 85% male) who underwent comprehensive cardiac CT consisted of calcium scoring, coronary CT angiography, stress perfusion CT and delayed enhancement CT were evaluated. Myocardial ECV was significantly greater in the hemodialysis group than in the control group (33.8 ± 4.7% versus 26.6 ± 2.9%; *P* < 0.0001). In the hemodialysis group, modest correlation was evident between myocardial ECV and left atrial volume index (*r* = 0.54; *P* = 0.01), while there was no correlation between myocardial ECV and other cardiac parameters including left ventricular mass index and severity of myocardial ischemia. Cardiac CT-based myocardial ECV may offer a potential imaging biomarker for myocardial fibrosis in HD patients.

## Introduction

The risk of cardiovascular mortality is increased in chronic kidney disease and hemodialysis (HD) populations^1,2^. The leading cause of cardiovascular death among HD patients is sudden cardiac death, however, the exact underlying arrhythmic mechanisms remain elusive^3^. A postmortem study of HD patients without coronary artery disease (CAD) showed that 98.3% had myocardial fibrosis, and the pattern of fibrosis was diffuse^4^. A recent study demonstrated that diffuse myocardial interstitial fibrosis, not replacement fibrosis, is associated with dispersion of ventricular repolarization in patients with hypertrophic cardiomyopathy^5^, which suggests the importance of including an assessment of interstitial fibrosis in risk stratification for ventricular arrhythmias. Traditionally, identification of myocardial fibrosis was performed by endomyocardial biopsy. However, this procedure is invasive and may lead to severe complications, while also suffering from sampling errors and interobserver discrepancies in interpretation^6,7^.

Cardiac magnetic resonance (CMR) can determine the extracellular volume fraction (ECV) of left ventricular (LV) myocardium by measuring T1 relaxation times before and after administration of gadolinium contrast, and has become a noninvasive standard reference of myocardial fibrosis^8–11^ Myocardial ECV is increased by myocardial fibrosis in a wide range of cardiac diseases, including cardiac sarcoidosis, cardiac amyloidosis, ischemic cardiomyopathy, hypertrophy cardiomyopathy and dilated cardiomyopathy^11^. An excellent correlation between myocardial ECV assessed by CMR and interstitial fibrosis determined by quantitative histopathology has been confirmed in a number of studies^8,9,12^. However, in patients with HD, low risk gadolinium-contrast medium for nephrogenic systemic fibrosis should only be administered with the smallest possible dose if contrast study is considered necessary for clinical management^13^.

Recently, several studies have demonstrated the potential of contrast enhanced cardiac computed tomography (CT) to assess myocardial fibrosis with myocardial ECV. Nacif et al. showed a good correlation between myocardial ECV values determined by CMR and cardiac CT (r = 0.82, *P* < 0.001)^14^. Moreover, Bandula et al. demonstrated a significant correlation between CT-derived myocardial ECV and histologic fibrosis as a percentage (r = 0.71, *P* < 0.001)^15^. Cardiac CT could thus offer a suitable approach for assessing myocardial fibrosis and coronary stenosis in a single study. However, whether or not ECV quantification by cardiac CT allows assessment of increased myocardial fibrosis in HD patients has not been investigated.

The present study sought to explore the ability of cardiac CT to evaluate the extent of interstitial myocardial fibrosis in HD patients by comparing CT-derived myocardial ECV between HD patients and propensity matched non-HD control subjects. In HD patients, we also examined the relationship between myocardial ECV and various parameters including LV mass index (LVMI), left atrial volume index (LAVI), and severity of myocardial ischemia.

## Materials and methods

### Study population

At our institution, a comprehensive cardiac CT consisted of coronary CT angiography (CTA), stress dynamic CT perfusion (CTP) and ECV measurement is considered for patients between 45 and 85 years old who are clinically referred for coronary CT angiography with moderate to high pretest likelihood for obstructive coronary artery disease. Between October 2013 and November 2016, a total of 402 patients underwent the comprehensive cardiac CT study. Among them, we consecutively recruited a total of 22 HD patients for this study. Of these, we excluded 2 patients who had undergone maintenance HD for < 12 months. Propensity score matched (age, sex, coronary risks and BMI) non-HD control subjects without any history of cardiovascular disease other than CAD were selected from the same cohort. The final study population comprised the 20 eligible HD patients and 20 propensity matched control subjects. This study was approved by the Clinical Research Ethics Review Committee of Mie University Hospital and performed in accordance with relevant guidelines and regulations. Written informed consent for study participation was obtained from all participants (including all control subjects).

### Image acquisition

Comprehensive cardiac CT examinations were performed using a second- or third-generation dual-source CT (SOMATOM Definition Flash or SOMATOM Force, Siemens Healthcare, Forchheim, Germany). A comprehensive cardiac CT protocol has been described in detail in elsewhere^16^, but in brief, the protocol included unenhanced CT (for coronary artery calcium scoring and calculation of ECV), stress dynamic CTP (evaluation of myocardial ischemia), coronary CTA and delayed-phase CT (for evaluation of delayed enhancement and calculation of ECV).

Stress dynamic CTP was performed with adenosine triphosphate administration and injection of 40 mL (an iodine concentration of 370 mgI/mL) of iopamidol (Iopamiron 370; Bayer-Schering Pharma, Berlin, Germany) at a flow rate of 5 mL/s^17^. Dynamic data sets were acquired for 30 s via an electrocardiographically triggered axial scan mode, repeated at 2 alternating table positions. Tube voltage was set at 80 kV and 70 kV with the second- and third-generation dual-source CT, respectively, and tube current was determined using an automatic exposure control system with a quality reference of 350 mAs/rot at 120 kV for the second-generation and 300 mAs/rot at 80 kV for the third-generation scanner. A standard prospective CTA was performed 10 min after stress dynamic CTP with injection of contrast medium at 0.84 mL/kg over 12 s (26 mgI/kg/s). Tube voltage was 2 × 100 kV or 80 kV in the second-generation scanner and 2 × 80 kV or 70 kV in the third-generation scanner, and tube current was determined using the automatic exposure control system with a quality reference of 350 mAs/rot at 120 kV in the second-generation scanner and 300 mAs/rot at 120 kV in the third generation scanner. Gantry rotation time was 0.28 s in the second-generation scanner and 0.25 s in the third-generation scanner.

Seven minutes after coronary CTA, end-systolic delayed-phase images were acquired by electrocardiographically triggered axial scan at two alternating table positions^18^. Tube voltage was 80 kV and tube current was 370 mA for the second-generation scanner, and was automatically determined by the automatic exposure control system with the quality reference of 580 mAs/rotation at 80 kV for the third-generation scanner. Pre-contrast CT was performed using the same acquisition protocol applied for delayed-phase CT. The time interval between stress dynamic CTP and delayed-phase CT was approximately 20 min.

### Image analysis

#### Delayed enhancement and ECV

**Figure 1:**
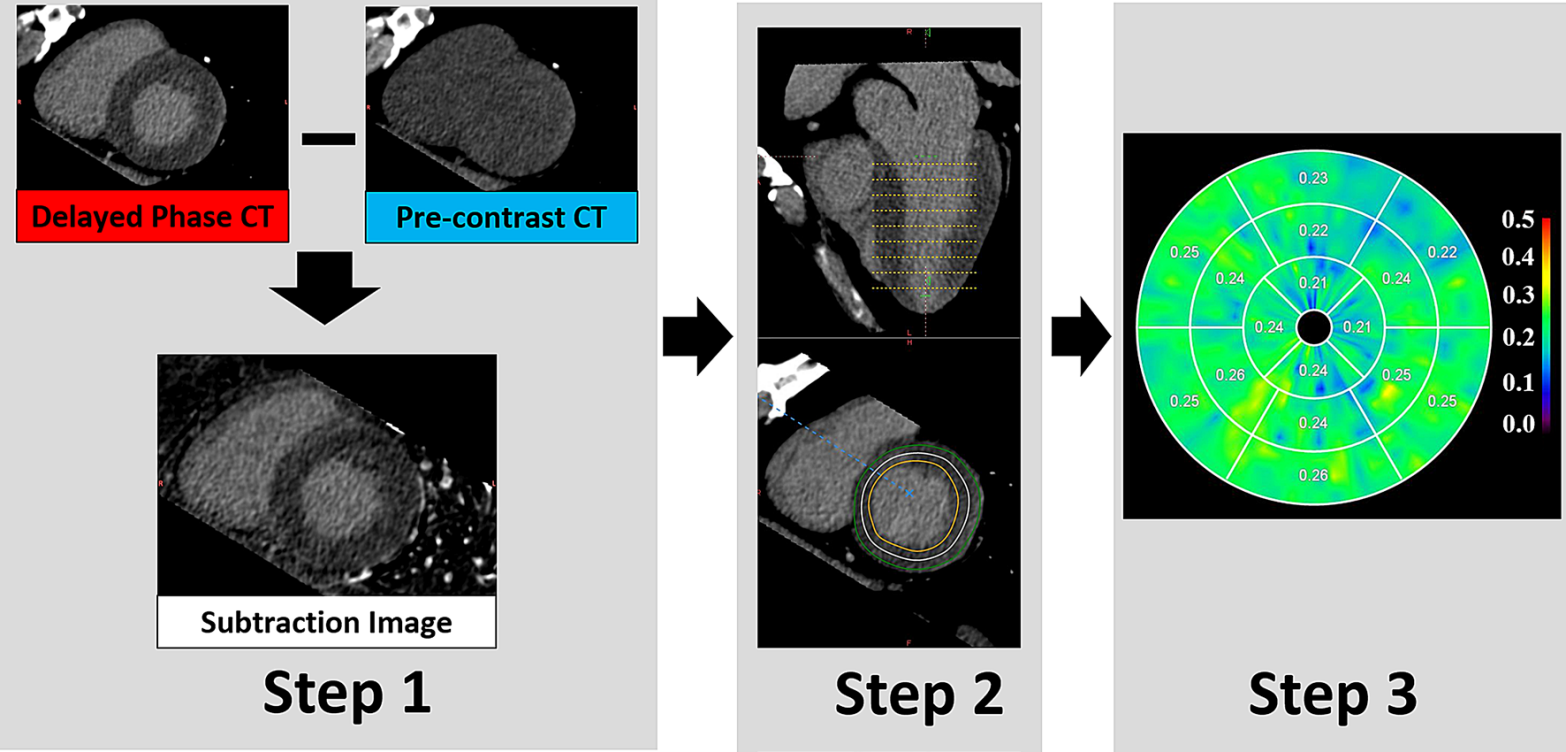
The process of calculating myocardial extracellular volume (ECV). Step 1: Generation of subtraction image. Subtraction images are generated from pre-contrast and delayed-phase CT by using an automatic three-dimensional non-rigid image registration. Step 2: Determination of left ventricular (LV) axis and segmentation of LV myocardium (tracing of endocardial and epicardial border). This step is also automated in the software with function of manually correction. A region of interest is placed in the LV cavity on the subtraction image to obtain ΔHUb. Step 3: Generation of ECV polar map. Entering the hematocrit value gives the ECV values for each segment in a polar map. In the polar map, ECV values are displayed as fractions.

The presence of ischemic and non-ischemic enhancement was visually evaluated on delayed-phase CT by two observers, including a radiologist with > 10 years of experience in cardiac CT and CMR. Discrete subendocardial or transmural delayed enhancement in a coronary distribution was considered an ischemic pattern, while patchy or diffuse enhancement in the midwall or subepicardium with sparing of the subendocardium was considered non-ischemic^19,20^. Myocardial ECV was calculated using commercial software (CT myocardial ECV analysis; Ziosoft, Tokyo, Japan) employing the following equation: ECV = (ΔHUm/ΔHUb)·(1 − Hct), where ΔHUm is the change in myocardial CT attenuation, in Hounsfield units (HU), ΔHUb is the change in CT attenuation of the blood, and Hct is the hematocrit^21^ (Supplementary Material Online 1). In this software, automatic three-dimensional non-rigid registration of the myocardium is performed between unenhanced and delayed phase CT to generate subtraction image^22^. Then, change in CT attenuation (ΔHU) was obtained on the subtraction image (Fig. 1). Subendocardial and subepicardial borders were contoured manually by two independent observers to allow assessment of interobserver reproducibility. The software finally produces a polar map showing 16 American Heart Association myocardial segments with the mean ECV value for each segment. In this study, ECV values in segments with ischemic pattern were excluded from further analysis.

#### Stress dynamic CTP

Stress dynamic CTP images were postprocessed using commercially available perfusion software (syngo VPCT body; Siemens Healthcare). Myocardial blood flow (MBF) was estimated using a dedicated parametric deconvolution technique, based on a 2-compartment model of the intravascular and extravascular spaces^23^. Maximum slopes of time-attenuation curves fitted for every voxel were used to generate an MBF map of 3-mm thickness and 1-mm increments. Polygonal regions of interest that measured 1–2 cm^2^ were placed within each of the 16 American Heart Association myocardial segments. Global MBF was calculated as the mean of the 16 segments. Severity of myocardial ischemia was evaluated using a summed stress score (SSS)^17^. A normalized MBF value was calculated as an MBF value in each segment divided by the highest MBF value within the 16 segments on an MBF map, then SSS was calculated by adding scores of all segments using a 5-point scale based on normalized MBF values: 0 = normal (> 0.75); 1 = mildly abnormal (≤ 0.75, > 0.675); 2 = moderately abnormal (≤ 0.675, > 0.60); and 3 = severely abnormal (≤ 0.60).

#### Assessment of coronary artery

The amount of coronary artery calcium was quantified from unenhanced CT using the Agatston scoring method^24^. Coronary CTA images were visually evaluated for the presence of stenosis in segments with a reference diameter ≥ 1.5 mm as the consensus decision of two observers. Severity of CAD was ranked using the Coronary Artery Disease Reporting and Data System (CAD-RADS): 0, no atherosclerotic change; 1, 1–24% stenosis; 2, 25–49%; 3, 50–69%; 4A, 70–99% in 1 to 2 vessels; 4B, 70–99% in 3 vessels or ≥ 50% in the left main coronary artery; or 5, 100%. Obstructive CAD was defined as stenosis ≥ 50% in ≥ 1 vessel (CAD-RADS ≥ 3)^25^.

#### LV volume and function, left atrial volume

LV volumes and LV ejection fraction (EF) were echocardiographically calculated using the modified Simpson method from apical 4- and 2-chamber views. LV systolic dysfunction was defined as an LVEF less than 50%^26^. Echocardiography was performed in the supine position using a Vivid7 Dimension cardiovascular ultrasound system (GE Vingmed Ultrasound AS, Horten, Norway). LV mass was obtained from coronary CTA using commercially available software (Syngo.CT Cardiac Function; Siemens Healthcare).

Left atrial volume was measured from CT datasets (40–75% of the R-R interval before opening of the atrioventricular valves) on a three-dimensional CT workstation (Ziostation2; Ziosoft, Tokyo, Japan) with the biplane area length method^27^.

### Statistical analysis

Normality was assessed using the Shapiro–Wilk test. Values are expressed as mean ± standard deviation, or median (interquartile range) as appropriate. Values were compared between the two groups by independent t tests and Mann–Whitney *U* tests. Categorical data were assessed using the chi-square test or Fisher’s exact test, as appropriate. Correlations within each group between continuous indices were assessed using Pearson’s and Spearman’s correlation coefficients for parametric and nonparametric data, respectively. In a previous study^22^, ECV within each subject group was normally distributed with standard deviation of 5 percent points. Assuming a true difference of 5 percentage points between the population means of the HD and control groups, we will need to study 17 HD patients and 17 control subjects to be able to reject the null hypothesis that the population means of the HD and control groups are equal with the power of 0.80 and the Type I error probability of 0.05. Thus, the sample size of 20 per group in this study was considered sufficient for the purpose. Propensity scores for the likelihood of coronary artery disease (age, sex, hypertension, dyslipidemia, diabetes mellitus, current smoking, previous percutaneous coronary intervention, history of myocardial infarction, and BMI) were obtained by logistic regression. Matching was made on logit-transformed propensity scores matched to the nearest neighbor in a 1:1 fashion with a caliper of 0.05. A blinded list of mixed HD patients and control subjects in a random order was used for CT image analysis. Two independent observers measured ECV to assess inter-observer reproducibility. Furthermore, one observer measured ECV twice with a washout period of 3 months to determine intra-observer reproducibility. The inter- and intra-observer reproducibility of the myocardial ECV measurement were tested by calculating mean bias and 95% limits of agreement (confidence intervals) from Bland–Altman analyses, the coefficient of variation (CV), and interclass correlation coefficient (ICC). Values of P < 0.05 were considered significant. The analyses were performed using SPSS software (version 23; SPSS, Chicago, IL).

## Results

### Baseline patient characteristics

Demographic data for 20 HD patients and 20 propensity matched non-HD control subjects are shown in Table 1. No differences in age, sex, body mass index, blood pressure, drug and medical history, history of myocardial infarction, and history of CAD were identified between groups. Hematocrit was lower in the HD group than in the control group. Mean dose-length product for the whole comprehensive cardiac examination was 806.7 ± 288.7 Gy cm, corresponding to an estimated effective dose of 11.3 ± 4.0 mSv. Mean radiation dose for ECV measurement (total of pre-contrast CT and delayed-phase CT) was 3.4 ± 0.6 mSv. The iodinated contrast dose was 40 mL for stress dynamic CTP (an iodine concentration of 370 mgI/mL) and 0.84 mL/kg body weight for CTA. In addition, 10 mL of contrast medium was used to determine the timing of CTA acquisition (test bolus). No additional contrast was injected after CTA before delayed-phase CT. The cumulative dose of injected contrast agent per patient was 102.6 ± 9.9 mL in this study. All patients were in sinus rhythm and free of valvular disease.

**Table 1:** Demographic data of HD patients and control subjects.

	HD (n = 20)	Control (n = 20)	*P* value
Age (years)	67.4 ± 9.6	66.3 ± 9.1	0.71
Male (n, %)	16 (80%)	17 (85%)	0.68
BMI (kg/m^2^)	23.4 ± 3.8	23.7 ± 2.3	0.73
BSA (m^2^)	1.7 ± 0.2	1.7 ± 0.2	0.44
Dialysis vintage (months)	124.2 (48–156)	–	
Hematocrit* (%)	33.4 ± 5.0	41.0 ± 3.9	< 0.0001
SBP* (mmHg)	141.7 ± 20.8	141.3 ± 14.2	0.94
DBP (mmHg)	68.3 ± 15.7	75.0 ± 8.4	0.10
HR (beats/min)	68.5 ± 10.6	63.3 ± 7.7	0.09
eGFR (ml/min/1.73 m^2^)	–	71.7 ± 15.6	
**Medical and drug history**			
Hypertension (n, %)	18 (90%)	16 (80%)	0.38
Diabetes (n, %)	11 (55%)	10 (50%)	0.75
Dyslipidemia (n, %)	9 (45%)	11 (55%)	0.53
History of CAD (n, %)	7 (35%)	8 (40%)	0.74
History of MI (n, %)	3 (15%)	4 (20%)	0.63
ACEi/ARB (n, %)	9 (45%)	8 (40%)	0.75
Calcium channel blocker (n, %)	12 (60%)	10 (50%)	0.53
Alpha-blocker (n, %)	1 (5%)	1 (5%)	1.00
Beta-blocker (n, %)	9 (45%)	5 (20%)	0.19
Diuretic (n, %)	3 (15%)	3 (15%)	1.00
Statin (n, %)	7 (35%)	11 (55%)	0.20
Antiplatelet (n, %)	16 (80%)	13 (65%)	0.29
Antidiabetic drugs (n, %)	4 (20%)	7 (35%)	0.29
Insulin (n, %)	4 (20%)	2 (10%)	0.38
Erythropoietin (n, %)	20 (100%)	–	
**Etiology of renal disease**			
Diabetic nephropathy (n, %)	10 (50%)		
Nephrosclerosis (n, %)	6 (30%)		
Glomerulonephritis (n, %)	2 (10%)		
Polycystic kidney disease (n, %)	1 (5%)		
Cholesterol crystal embolism (n, %)	1 (5%)		
Propensity score	0.129 ± 0.103	0.120 ± 0.106	0.78

All data are shown as mean ± standard deviation, median (interquartile range), or number of participants (percentage), as appropriate.

*BMI* body mass index, *BSA* body surface area, *SBP* systolic blood pressure, *DBP* diastolic blood pressure, *HR* heart rate, *eGFR* estimated glomerular filtration rate, *CAD* coronary artery disease, *MI* myocardial infarction, *ACEi* angiotensin-converting enzyme inhibitor, *ARB* angiotensin receptor blocker.

*P < 0.05.

### Coronary artery calcium score, coronary CTA, stress dynamic CTP and delayed-phase CT

Table 2 shows the results of coronary artery calcium scoring, coronary CTA, stress dynamic CTP and delayed-phase CT in the two groups. Total coronary artery calcium score was significantly higher in the HD group than that of control group (1993.1 ± 2,465.6 vs. 277.6 ± 406.5; *P* = *0.009*). However, there was no difference in severity of CAD as classified by CAD-RADS category, global MBF (107.0 ± 40.4 vs. 120.9 ± 28.8; *P* = *0.22*) or SSS (8.8 ± 9.1 vs. 6.4 ± 6.4; *P* = *0.66*). As for delayed-phase CT, 7 HD patients (22 of the 320 segments) and 8 control subjects (34 of the 320 segments) showed ischemic delayed enhancement, while none demonstrated non-ischemic delayed enhancement. All segments with ischemic delayed enhancement were excluded from ECV analysis.

**Table 2:** Imaging results of coronary artery calcium scoring, coronary CTA, stress dynamic CT perfusion, and delayed-phase CT.

	HD (n = 20)	Control (n = 20)	*P* value
**Coronary calcium scoring**			
Total*	1,993.1 ± 2,465.6	277.6 ± 406.5	0.009
**Coronary CTA**			
CAD-RADS 0–2 (n, %)	6 (30%)	11 (55%)	0.11
CAD-RADS 3 (n, %)	3 (15%)	5 (25%)	0.43
CAD-RADS 4A (n, %)	4 (20%)	2 (10%)	0.38
CAD-RADS 4B (n, %)	1 (5%)	0 (0%)	0.31
CAD-RADS 5 (n, %)	6 (30%)	2 (10%)	0.11
Obstructive CAD (n, %)	14 (70%)	9 (45%)	0.11
Single-vessel disease (n, %)	7 (35%)	5 (25%)	0.49
Multivessel disease (n, %)	7 (35%)	4 (20%)	0.29
**Stress dynamic CTP**			
Global MBF (mL/100 mL/min)	107.0 ± 40.4	120.9 ± 28.8	0.22
SSS	8.8 ± 9.1	6.4 ± 6.4	0.66
SSS ≥ 4 (n, %)	13 (65%)	11 (55%)	0.52
SSS ≥ 8 (n, %)	8 (40%)	5 (25%)	0.31
SSS ≥ 12 (n, %)	7 (35%)	3 (15%)	0.14
**Delayed-phase CT**			
Ischemic pattern (n, %)	7 (35%)	8 (40%)	0.74
Non-ischemic pattern (n, %)	0 (0%)	0 (0%)	1.00
Myocardial ECV (%)*	33.8 ± 4.7	26.6 ± 2.9	< 0.0001

*CAD-RADS* Coronary Artery Disease-Reporting and Data System, *CAD* coronary artery disease, *CTP* computed tomography perfusion, *MBF* myocardial blood flow, *SSS* summed stress score, *MI* myocardial infarction, *ECV* extracellular volume fraction.

*P < 0.05.

### Myocardial ECV, LV mass, function and left atrial volume

**Figure 2:**
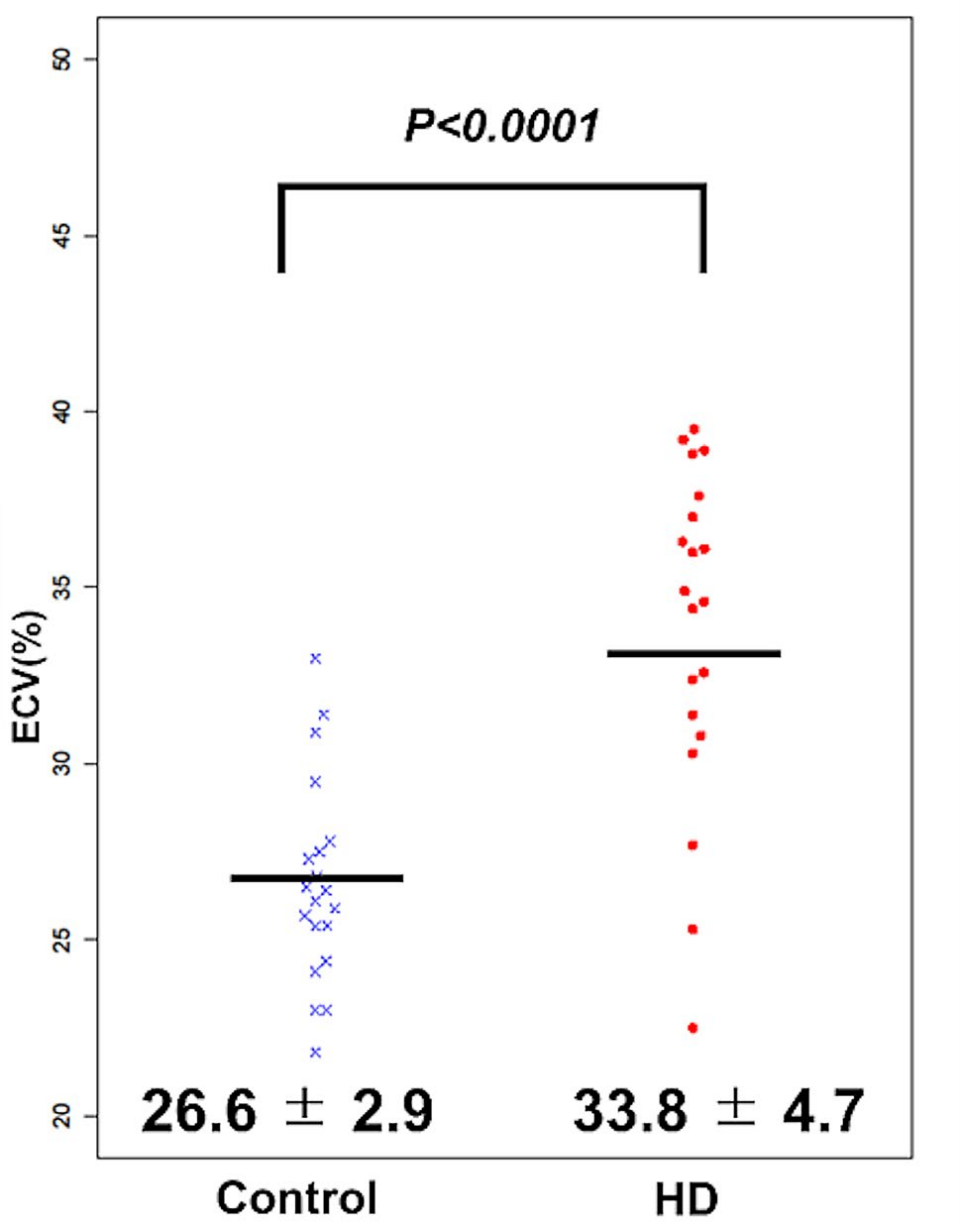
Bee swarm plot comparing myocardial extracellular volume (ECV) in hemodialysis (HD) group and control group.

**Figure 3:**
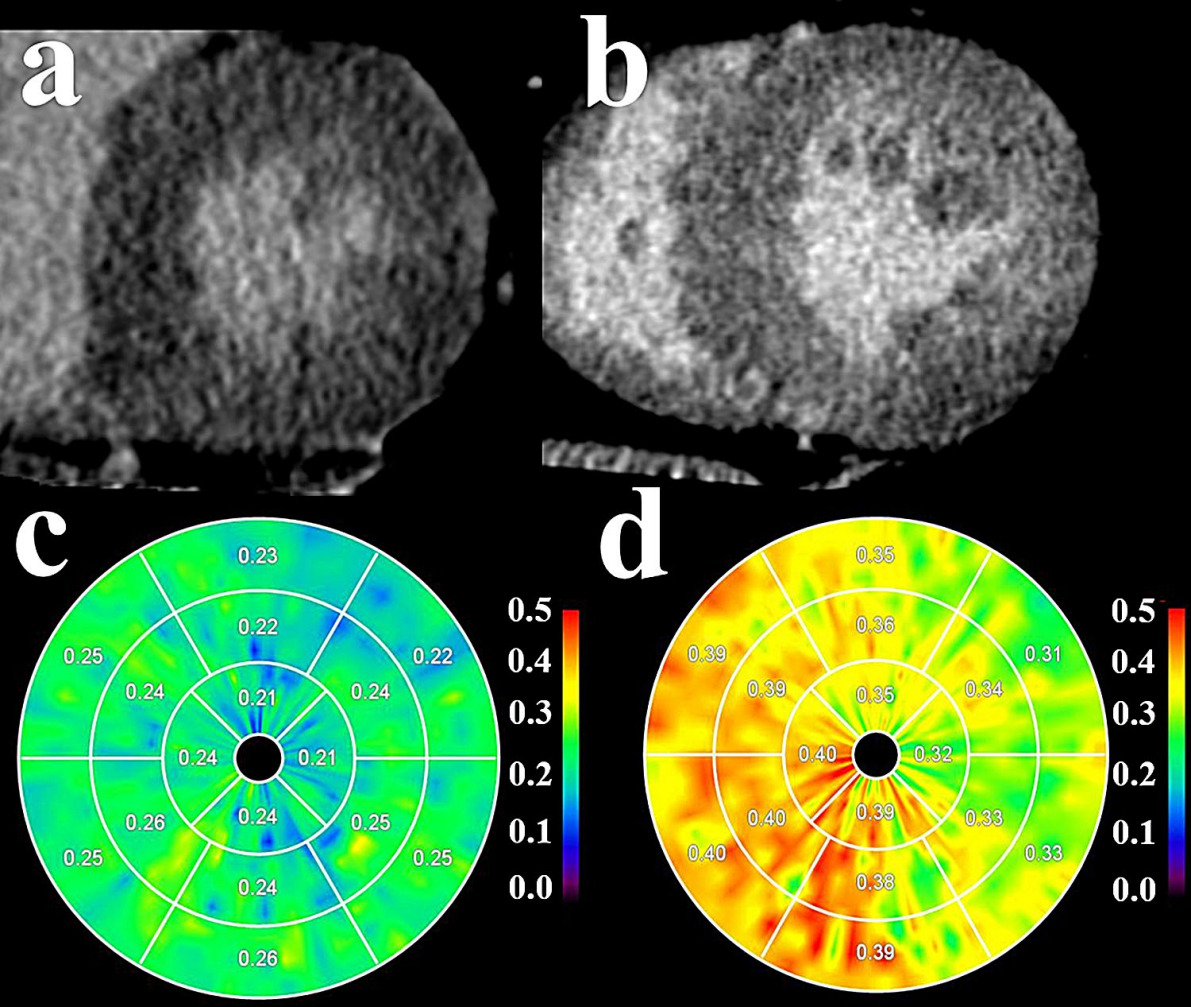
Delayed enhancement images (**a**, **b**) and myocardial extracellular volume (ECV) polar maps (**c**, **d**) of a control subject and a hemodialysis (HD) patient. No focal area of delayed enhancement is detected in the control subject (**a**) or HD patient (**b**). The myocardial ECV polar map of the control subject (**c**) shows normal myocardial ECV. The myocardial ECV polar map of the HD patient (**d**) shows higher ECV, indicative of advanced myocardial fibrosis that is undetectable from the delayed enhancement image (**b**). In the polar maps, ECV values are displayed as fractions.

Myocardial ECV was significantly greater in the HD group (33.8 ± 4.7%) than in the control group (26.6 ± 2.9%; *P* < 0.0001) (Fig. 2). The mean LVEF was 57.1 ± 13.3% in HD group and 64.6 ± 5.7% in control group (*P* = 0.15). Although none showed overt heart failure, LV systolic dysfunction was observed in 7 patients (35%) in the HD group and 1 patient in the control (5%; *P* = 0.02). However, even in a subgroup of patients without LV systolic dysfunction, myocardial ECV was significantly higher in 13 HD patients compared to 19 control subjects (33.5 ± 5.1% vs 26.3 ± 2.7%, P = 0.002). The examples of myocardial ECV map are shown in Fig. 3. LVMI was significantly greater in the HD group (90.9 ± 28.9 g/m^2^) than in the control group (64.5 ± 11.0 g/m^2^; *P* < 0.0001). LAVI was significantly increased in the HD group than in the control group (51.9 ± 22.4 mL/m^2^ vs 30.2 ± 10.4 mL/m^2^; *P* < 0.0001) (Table 3). The segmental variation of myocardial ECV was shown in Supplementary Material Online 2.

**Table 3:** Cardiac parameters of HD patients and control subjects.

	HD (n = 20)	Control (n = 20)	*P* value
LVEDV (mL)	105.0 ± 40.6	99.2 ± 19.7	0.74
LVEDVI (mL/m^2^)	64.6 ± 26.9	59.2 ± 14.4	0.84
LVESV (mL)	48.4 ± 30.6	36.3 ± 11.5	0.55
LVESVI (mL/m^2^)	29.7 ± 19.9	21.6 ± 7.5	0.55
LVEF (%)	57.1 ± 13.3	64.6 ± 5.7	0.15
LVM* (g)	148.0 ± 45.2	107.1 ± 18.8	< 0.001
LVMI* (g/m^2^)	90.9 ± 28.9	64.5 ± 11.0	< 0.0001
LAV* (ml)	85.4 ± 41.3	50.7 ± 16.8	< 0.0001
LAVI* (ml/m^2^)	51.9 ± 22.4	30.2 ± 10.4	< 0.0001

All data are shown as mean ± standard deviation.

*HD* hemodialysis, *ECV* extracellular volume fraction, *LAV* left atrial volume, *LAVI* left atrial volume index, *LVM* left ventricular mass, *LVMI* left ventricular mass index, *LVEF* left ventricular ejection fraction, *LVEDV* left ventricular end-diastolic volume, *LVEDVI* left ventricular end-diastolic volume index, *LVESV* left ventricular end-systolic volume, *LVESVI* left ventricular end-systolic volume index, *DLP* dose-length product.

*P < 0.05.

### Correlation of myocardial ECV in HD group with cardiac and HD parameters

**Figure 4:**
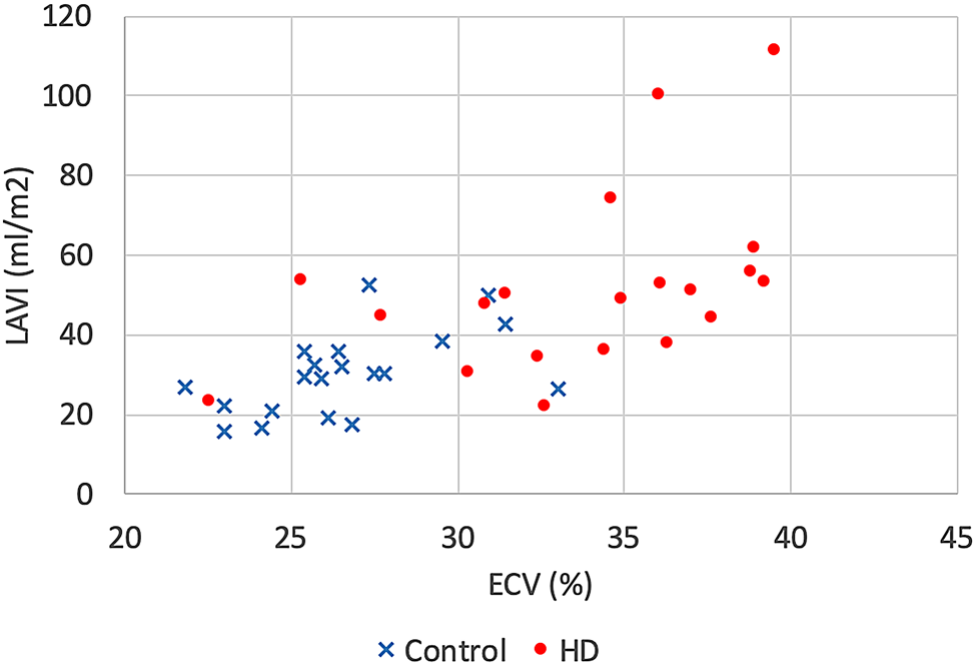
Relationship between extracellular volume (ECV) and left atrial volume index (LAVI) in hemodialysis (HD) group and control group.

Correlations between myocardial ECV and various parameters in HD group are shown in Table 4. Myocardial ECV correlated significantly with LAVI both in HD group (*r* = 0.54; *P* = 0.01) and in the control (r = 0.56; *P* = 0.01) (Fig. 4). Myocardial ECV did not show significant association with SSS, CAD-RADS, LVMI, or LVEF. No correlation was identified between myocardial ECV and interdialysis body weight gain (difference between weight at the end of dialysis and weight at the time of scanning), ultrafiltration volume, or dialysis vintage.

**Table 4:** Correlation of myocardial ECV in HD patients.

Parameters	Myocardial ECV	
	r	P
Age	0.18	0.44
BMI	0.07	0.76
SBP	0.22	0.35
DBP	0.06	0.81
SSS	− 0.36	0.12
MBF	0.12	0.61
CAD-RADS	0.24	0.31
Coronary artery calcium score	0.36	0.23
LVEF	− 0.18	0.44
LVEDVI	− 0.06	0.81
LVESVI	0.16	0.51
LVMI	0.33	0.16
LAVI*	0.54	0.01
QRS duration	− 0.07	0.78
Dialysis vintage	0.06	0.80
Ultrafiltration volume	0.06	0.80
Interdialysis BW gain	− 0.17	0.50

*ECV*: extracellular volume fraction; *BMI*: body mass index; *SBP*: systolic blood pressure; *DBP* diastolic blood pressure, *SSS* summed stress score, *MBF* myocardial blood flow, *CAD-RADS* Coronary Artery Disease-Reporting and Data System, *LVEF* left ventricular ejection fraction, *LVEDVI* left ventricular end-diastolic volume index, *LVESVI* left ventricular end-systolic volume index, *LVMI* left ventricular mass index, *LAVI* left atrial volume index, *BW* body weight.

*P < 0.05.

### Inter- and intra-observer reproducibility

The mean inter-observer difference was 0.48 ± 1.50% (95% limits of agreement: − 3.42 to 2.46%), while the mean intra-observer difference was 0.28 ± 1.37 (95% limits of agreement: − 2.97 to 2.41%). The CV for the inter-observer and intra-observer measurements of myocardial ECV was 3.20% and 2.98%, respectively. The ICC for the inter-observer and intra-observer measurements of myocardial ECV were 0.962 (95% confidence interval: 0.905 to 0.985) and 0.966 (95% confidence interval: 0.914 to 0.987).

## Discussion

In the current study, we investigated the extent of diffuse myocardial fibrosis by comparing myocardial ECV derived from cardiac CT between HD patients and control subjects for the first time. The main findings of our study were: (1) myocardial ECV was significantly greater in HD patients than in control subjects; (2) there was a significant correlation between myocardial ECV and LAVI in HD group.

By simply quantifying the interstitial presence of contrast medium relative to the plasma, ECV is well suited to measure interstitial expansion occurring with fibrosis in the absence of interstitial edema^28^. Compared to native T1 mapping by CMR, which potentially allows quantification of myocardial fibrosis in HD patients without use of contrast media^29,30^, ECV generally shows better agreement with histologic fibrosis. This is because native T1 is affected by more confounders (e.g. lipids, iron, intracellular edema) than ECV^28^. Additionally, myocardial ECV derived from cardiac CT had excellent inter- and intra-observer reproducibility in this study, which enhaced the validity and reliability of this study.

Histological studies have shown that diffuse (not focal) myocardial fibrosis was found in HD patients^4,31,32^. Analogous to these previous reports, the greater myocardial ECV of the HD group (33.8 ± 4.7%) in the present study would represent a development of myocardial fibrosis, whereas ECV of the controls (26.6 ± 2.9%) is within the normal range. Since it is well documented that ECV is elevated in patients with heart failure with reduced EF^33^ and there were more patients with LVEF < 50% in the HD group than in the control group, we performed a subgroup analysis that excluded patients with LVEF < 50% and confirmed that ECV was higher in the HD group. Of note, we found a significant correlation between myocardial ECV and LAVI in HD patients. Elevated ECV is reported to be a major contributor to the impaired LV relaxation and stiffness^34^. With increased LV stiffness, left atrial pressure rises to maintain adequate ventricular filling, and the increased atrial wall tension leads to subsequent left atrial enlargement. Our results showing the correlation between myocardial ECV and LAVI is consistent with this pathophysiological cascade and demonstrates the accuracy for the quantification of ECV in our study.

However, several factors potentially affecting myocardial ECV value should be considered when interpreting the results. First, the presence and extent of atherosclerosis in the coronary circulation may be related with increased myocardial ECV in HD patients. Indeed, HD patients exhibited higher calcium scores in the current study. However, we found no significant difference in the severity of obstructive CAD or the severity of myocardial ischemia between HD and control patients. While the effect of chronic myocardial ischemia appears limited in this study, a large-scale study is warranted to explore the effect of chronic myocardial ischemia.

Second, whether fluid shifts affect myocardial ECV in HD patients remains an issue of concern since this is crucial to define the reliability of myocardial ECV as a potential marker of myocardial fibrosis among HD patients. However, a recent cardiac MR study by Graham-Brown et al. showed that changes in fluid status among HD patients did not affect native T1 signal, which is theoretically more sensitive to fluid status than myocardial ECV^29^. In our study, interdialysis body weight gain and ultrafiltration volume, representative of changing fluid status, showed no correlations with myocardial ECV. Dialysis vintage also did not show correlation with myocardial ECV. In this regard, a potential explanation is that myocardial fibrosis may correlate with duration of impaired renal dysfunction^35^. However, the accurate determination of the duration of renal dysfunction is generally difficult because determining the beginning of renal impairment is impossible.

### Limitations

This study has some limitations that require acknowledgement. First, myocardial ECV data in our study were derived from a relatively small cohort from a single center. A large-scale study is warranted to validate the results of our study. Second, histologic validation was not performed. Subjects in this study were not eligible for endomyocardial biopsy. However, a previous study showed myocardial ECV determined by cardiac CT correlated with histologic quantification of myocardial fibrosis^15^. Moreover, the histological studies have demonstrated that HD patients showed a larger extent of diffuse myocardial fibrosis^4,19^. Third, although total radiation dose (11.3 ± 4.0 mSv) applied in this study was acceptable, combining ECV module and stress dynamic CTP with coronary CTA increased the doses of ionizing radiation and contrast agent compared with coronary CTA alone. Continuous effort to reduce radiation exposure and contrast agent is required. Forth, scan-rescan reproducibility, which is necessary to understand the potential usefulness of ECV in the serial assessment, was not evaluated in this study. Although a recent study showed a high repeatability of CT-derived ECV in patients with suspected coronary artery disease who underwent serial assessment of ECV with a median interval of 5.1 months^36^, future investigation of repeatability of ECV in HD patients is awaited. Finally, this study did not address clinical outcomes, further studies are warranted to reveal the relationship between myocardial ECV and future cardiac events.

## Conclusion

Cardiac CT-based myocardial ECV offers a potential imaging biomarker for myocardial fibrosis in HD patients. Further work is warranted regarding the relationship between myocardial ECV and the development of arrhythmia and heart failure in HD patients.

## Supplementary information

Supplementary Information.

## Abbreviations

HD: Hemodialysis
CAD: Coronary artery disease
CMR: Cardiac magnetic resonance
ECV: Extracellular volume fraction
LV: Left ventricular
CT: Computed tomography
LVMI: Left ventricular mass index
LAVI: Left atrial volume index
CTA: Computed tomography angiography
CTP: Computed tomography perfusion
MBF: Myocardial blood flow
SSS: Summed stress score
CAD-RADS: Coronary artery disease-reporting and data system
LVEF: Left ventricular ejection fraction
