# Supplementary material for: Quantification of extracellular volume fraction by cardiac computed tomography for noninvasive assessment of myocardial fibrosis in hemodialysis patients
Source: Sci Rep. 2020 Sep 21;10:15367. doi: 10.1038/s41598-020-72417-5 (PMC7506012; doi:10.1038/s41598-020-72417-5)
Supplement: Supplementary file 1 — Supplementary Information. [file 41598_2020_72417_MOESM1_ESM.docx]

SUPPLEMENTARY FILE

Quantification of extracellular volume fraction by cardiac computed tomography for noninvasive assessment of myocardial fibrosis in hemodialysis patients

Akimasa Yamada, MD^1^, Kakuya Kitagawa, MD, PhD^2*^, Satoshi Nakamura, MD, PhD^1^, Masafumi Takafuji, MD^1^, Yoshitaka Goto, MD, PhD^1^, Ryuji Okamoto, MD, PhD^3^, Kaoru Dohi, MD, PhD^3^, Hajime Sakuma, MD, PhD^1^

Departments of ^1^Radiology and ^3^Cardiology, Mie University Hospital

Departments of ^2^Advanced Diagnostic Imaging, Mie University Graduate School of Medicine

Page 1 of 3

**Supplement 1: Theoretical background of calculations of extracellular volume fraction.**

The concentration of contrast in the plasma (C_p_) can be given as the concentration of contrast in the whole blood (C_b_) divided by 1- hematocrit (Hct):

C_p_ = C_b_ / (1-Hct) (1)

It is assumed the concentration of contrast reaches an equilibrium state where the concentration in the extravascular extracellular space (C_e_) equals the plasma concentration (C_p_):

C_p_ = C_b_ / (1-Hct) = C_e_ (2)

The concentration of contrast in the tissue (C_t_) can be expressed as the weighted average of the concentrations in the extravascular extracellular and the plasma spaces:

C_t_ = VeC_e_ + VpC_p_ (3)

Ve and Vp denote the volume fractions of the extravascular extracellular and the plasma spaces, respectively. The total of Ve and Vp equals to tissue extracelluar volume fraction (ECV).

By combining equation (2) and (3):

Ve + Vp = C_t_ (1-Hct)/C_b_  (4)

This equation indicates that ECV (= Ve + Vp) can be calculated as ratio of C_t_ divided by C_b_ corrected by HCT. In CT, the ratio of C_t_ divided by C_b_ can be simply given as the ratio of increase of myocardial HU divided by increase of LV blood HU because CT HU shows linear correlation with concentration of the iodinated contrast medium.

Consequently, myocardial ECV is represented by:

[ΔHU_myocradium_ (1-Hct)/ΔHU_LV blood_ ] ×100 (%) Page 2 of 3

**Supplement 2: Regional variation of myocardial extracellular volume fraction. Values are presented as mean ± standard deviation.**

Page 3 of 3
